# Effects of repetitive head impacts from a single season on the cognitive functioning of youth male soccer players

**DOI:** 10.1371/journal.pone.0329329

**Published:** 2025-07-31

**Authors:** Rachel S. Watson, Lauren Sergio, Haojie Mao, Jeffrey S. Brooks, James P. Dickey

**Affiliations:** 1 Faculty of Health Sciences, School of Kinesiology, Western University, London, Ontario, Canada; 2 Faculty of Health, School of Kinesiology and Health Science, York University, Toronto, Ontario, Canada; 3 Faculty of Engineering, Mechanical and Materials Engineering, Western University, London, Ontario, Canada; ASPIRE Academy for Sports Excellence, QATAR

## Abstract

Repetitive head impacts have long-lasting negative effects on the cognition of athletes. For example, repetitive head impacts accumulated by adult professional soccer players throughout their careers result in long-term negative consequences on cognition. However, these effects on youth soccer players have not been extensively studied and need to be further evaluated. The purposes of this study were to quantify head impact exposure in youth elite soccer and to examine the effects of repetitive head impacts on the cognitive function of youth male soccer players. A prospective cohort study of a single boys U13 soccer team of 18 players (12.9 ± 0.2 years of age) was completed throughout a single soccer season (five months in duration). Head impact frequency data were recorded using impact monitoring mouthguards worn during practices and matches and subsequently video-verified. Cognitive function was assessed using cognitive-motor integration (CMI) tasks conducted before the first season match, every subsequent four weeks, and after the last match. Peak path velocity, absolute error, reaction time, full path movement time, and the number of direction reversals were CMI task outcome measures. Over a single soccer season, 1089 head impacts occurred with more head impacts occurring in practices (62.7%) than matches (37.3%). Midfielders experienced the most head impacts during practices (53.6% of practice impacts), and defenders experienced the most head impacts during matches (47.8% of match impacts). Repetitive head impact exposure by position was associated with significant decreases in absolute error (*p < *0.001), increases in peak velocity *(p < *0.001), and increases in reaction time (*p < *0.001). Repetitive head impacts within a single soccer season were associated with measurable declines in some aspects of youth soccer players’ cognitive function. Therefore, coaches should aim to reduce the number of head impacts experienced by male youth soccer players with a specific focus on reducing head impact exposure during practices.

## Introduction

Soccer is the most popular sport in the world, with about 265 million players worldwide [1]. Of those players, 22 million are youth athletes under the age of 18 [1]. Soccer is the only sport where players intentionally use their unprotected heads to advance the ball. Soccer heading refers to a player’s use of their head to pass, shoot, or block the soccer ball while it is airborne. Within the sport of soccer, intentional headers account for about 90% of all head impacts [2]. Competitive youth soccer players under the age of 13 experience an average of 1.11 headers per match [3], with some players experiencing up to eight headers per match [4]. Additionally, the head impact rate per athlete exposure for male youth soccer players is more than three times that of male youth athletes in other non-contact sports such as basketball and lacrosse [5]. Head impact exposure in soccer can vary based on player position and header scenario [3,6]. In youth soccer, midfield players have the highest exposure rate than any other position on the field [3]. Midfielders experience 2.10 headers per match which is higher than defensive players and forwards who experience 1.66 headers and 1.34 headers per match, respectively [3]. Additionally, header scenario (e.g., throw-in, corner kick, drop kick) influences the magnitude and frequency of headers [6,7]. Passes in the air result in more headers than other header scenarios, especially when passes are longer than 20 meters [7–9]. Throw-ins result in the second most head impacts during matches [4].

Repetitive head impacts experienced by youth soccer players are associated with acute cognitive changes, including increased reaction time after a single soccer practice [10] and impaired reaction times after a single season compared to non-contact athletes [11]. Studies of youth and collegiate soccer players further demonstrate that cumulative seasonal repetitive head impact exposure correlates with neurophysiological alterations [3,6–9], underscoring the need to mitigate within-season risks. Although a single non-concussive soccer header does not result in noticeable symptoms of cognitive deficits, the accumulation of head impacts has severe negative effects on the cognition of adult athletes as seen by reduced psychomotor speed, verbal learning, and verbal memory [12]. Moreover, based on their stage of development, the accumulation of head impacts may have more serious consequences for youth athletes (younger than 18 years of age). During adolescence, executive function develops through the reorganization of grey matter in the brain [13]. This brain reorganization is sensitive to physiological changes [14], so repetitive head impacts during adolescence may disrupt executive function development and result in cognitive function impairments later in life [15], as worse executive function has been linked to poorer academic performance in young adults [16]. Youth athletes are also at an increased risk of head injury due to a greater ratio of their head-to-neck circumferences [17]. This discrepancy, and the subsequent reduction in neck strength causes a reduced ability to decrease head accelerations during collisions such as when a soccer ball collides with an athlete’s head [17,18].

Cognitive Motor Integration (CMI) is crucial for skilled performance of tasks of daily living and sports [19]. Cognitive motor integration tasks incorporate cognitive processing with physical activity, as seen in soccer when a player dribbles the ball while scanning the field for opponents. These tasks decouple vision and action where the visual cue is in a different plane from the movement required. Response inhibition, cognitive flexibility, and memory are crucial components of executive function [20] and are required when completing CMI tasks, especially when switching conditions from a standard to a non-standard condition when vision and action are decoupled. To successfully complete the CMI tasks, cognitive flexibility, memory, and response inhibition are required to disengage from the rule set of the previous task and to remember and engage with the rules of the new tasks, while inhibiting the tendency to produce an autonomic response as exercised with the standard condition. Additionally, attentional skills are essential for executive function [21], as well as to complete CMI tasks as efficiently and accurately as possible. Executive function and motor skills are positively associated [22,23], as such changes in performance on CMI tasks may reflect changes in executive function and an athlete’s motor skills. Dissociation between vision and movement results in visuomotor deficits in movement planning and executive function in adolescents with a concussion history [24]. Cognitive motor integration tasks can successfully differentiate between healthy and concussed adolescents and young adults with an accuracy of 70% [24] and 94% [25], respectively. Additionally, decreased performance on CMI tasks in which vision and action are decoupled are associated with decreased white brain matter structure in post-concussive patients [26]. Accordingly, CMI tasks may be a useful tool to measure cognitive processing changes and cognitive deficits due to repetitive head impacts during youth soccer.

Although concussions in sports are well-researched, most concussion research is conducted in adults and professional athletes which may not be generalizable to youth populations due to differences in brain development [27] and neck and shoulder strength [27,28]. Previous research has found that professional and amateur soccer players exhibit poorer scores on measures of neuropsychological testing due to repetitive head impacts when compared to controls [29,30]. However, research on the effects of repetitive head impacts in youth soccer are less conclusive [11,31], and warrant further exploration. As a result, the purpose of this study was to examine the relationship between repetitive head impacts and cognitive function in youth soccer players. This study will quantify head impact exposure by player position and header scenario in youth male soccer players throughout one soccer season. It is hypothesized that within a single soccer season, measurable cognitive function deficits will be seen in the youth players and these deficits will vary by player position with midfielders experiencing the largest deficits as they experience the most head impacts by player position [3].

## Methods

### Participants

A convenience sample of 18 male soccer players (12.9 ± 0.2 years of age) from an elite under-13 Ontario Player Development League team was used in this study (Team A). Participants from Team A were recruited in the month leading up to the summer playing season, beginning March 27^th^, 2023 and concluding on April 26^th^, 2023. Team B represented all opponents faced by Team A during matches (matches between opposing teams), aggregated into a single group to enable comparison of head impact exposure between Team A and typical in-match opponents. Data for Team B were derived exclusively from video recordings and observational coding during matches. Parents or guardians provided informed written consent and participants provided informed written assent. Players were ineligible to participate in the study if they exhibited concussion symptoms at enrollment or had not been medically cleared to return to activity from a previous concussion by a healthcare professional at the time of enrollment. No players were excluded based on these criteria. Players were also screened for history of concussion and mental health conditions. Players participated in four 75-minute soccer practices a week, one 90-minute competitive match per week, and one in-season tournament. Matches refer to formal competitions against external opponents. Practice sessions included structured drills, some of which involved heading (e.g., corner kicks, throw-ins, open play). Tournaments consisted of a series of three 70-minute competitive matches played over a single weekend. The soccer season was five months long, from May 2023 to the end of September 2023. Study procedures were approved by The University of Western Ontario Ethics Committee for Research on Human Subjects.

### Impact monitoring mouthguard instrumentation

Kinematic head impact metrics were collected from matches and practices using instrumented mouthguards. The Prevent boil-and-bite mouthguards were initially trialled but replaced after one week with custom-fit mouthguards due to a low proximity sensor sensitivity and to improve the fit of the mouthguard to the upper teeth [32]. A prior soccer study measured higher athletic performance and comfort ratings when wearing custom-fit mouthguards compared to standard boil-and-bite mouthguards [33]. Instrumented mouthguards have been previously used in youth soccer athletes to measure the kinematics of head impact exposure [34–36] and have been validated as accurate and sensitive measures of head impact data in youth [37]. A three-dimensional dental model for each player was generated using an iTero^TM^ dental scanner (Align Technology, Inc., Tempe, AZ) to create the Prevent custom mouthguard. The Prevent impact monitoring mouthguards (IMM; Prevent Biometrics, Edina, MN) are an independently validated head impact monitoring system [28,32]. The IMMs consisted of a tri-axial accelerometer and a tri-axial gyroscope which measured the linear and angular acceleration and velocity of all head impacts. Head impact events were recorded and stored on the IMM when linear acceleration magnitudes exceeded 5 g on any of the three axes. Recording of an event was triggered if the device’s proximity sensor determined the IMM was on the teeth.

### Video instrumentation

Head impact events were verified and classified using recorded match footage from a Veo video system. The Veo Cam 2 (Veo Sports Camera, Copenhagen, Denmark) uses an NVIDIA (NVIDIA, Santa Clara, CA) CUDA deep neural network graphics processing unit to analyze video footage in real-time. This artificial intelligence was used to track the ball and all on-field players, while key match events (e.g., corner kicks, free kicks, and goals) were time-stamped. The Veo Cam’s continuous ball tracking ensured full-field coverage and minimized missed footage, which reduced observational gaps that could occur with manual camera operation. All matches were recorded and footage was uploaded to the Veo online platform.

### Protocol

Impact-monitoring mouthguards were worn during all matches and practices throughout the season. Kinematic head impact data were stored on the IMMs. All season matches and practices with head impact drills (two of four weekly practices) were monitored by a member of the research team (RSW or JSB). For all head impacts – whether during matches or practice – the header scenario was recorded. However, the relative intensities of these scenarios were not quantified. Since IMMs may not capture every head impact experienced by players [32], observed data from matches and practices were recorded on data sheets and used to verify IMM head impacts. After each practice or match, the devices were connected via Bluetooth to upload all kinematic data to an online password-protected portal. Kinematic head impact data were amalgamated for analysis by downloading the date-specific data from the portal and exporting it to a master spreadsheet (*Microsoft Excel*, Version 16.83*)*. A single reviewer (RSW) with experience playing and coaching soccer evaluated match footage to maintain consistent implementation of head impact classification criteria. The time-stamped footage allowed the reviewer to efficiently locate and validate head impacts observed in real-time during matches. Head impact events for Teams A and B were categorized by player, position, and header scenario, following established methods for measuring head impacts in youth soccer [6]. The time-stamped head impacts from the IMMs were matched by the reviewer (RSW) to the corresponding match footage time stamp.

### Cognitive motor integration task instrumentation

**Fig 1:**
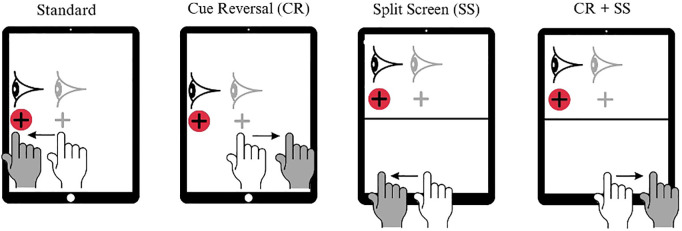
Diagram of the four CMI task conditions. The light grey eye, cursor, and white hand show the starting position for each trial. The dark grey hand and eye show the instructed movement for each task. The red circle shows the peripheral target.

Measures of cognitive functioning were collected using cognitive-motor integration (CMI) tasks completed on a Samsung Galaxy touchscreen tablet (Samsung Electronics, Suwon-si, South Korea) using the BrDI™ application (3MotionAI, Inc., Oakville, ON, Canada). This application has been used to measure cognitive motor integration outcomes in nonconcussed, concussed, and post-concussion patients [24–26,38]. Cognitive functioning was evaluated at six points during the season; at baseline before the first match of the season, after every four regular season matches, and the last evaluation occurred after the last season match. Each CMI task consisted of four conditions (one standard, three non-standard) in a fixed order and each condition contained 20 trials (Fig 1). In the standard condition, the player held their finger in the center of the tablet and, upon the random appearance of a target, moved a cursor on the screen with their finger to the target as fast as possible, holding it there for 500 ms. This duration was selected to operationalize sustained spatial motor control and accuracy, distinguishing deliberate actions from transient or imprecise movements. This condition measured baseline visuomotor coordination, such as passing to a teammate. There was an even distribution across the four target positions (up, down, left, and right; 38 mm center to target distance). The second non-standard condition was a cue reversal condition which required the player to move their finger in the opposite direction once the target appeared on the screen, evaluating inhibitory control and adaptive decision-making and mirroring scenarios where players must suddenly redirect play to avoid opponents. The third condition was a non-standard split-screen condition where the target and cursor appeared on the top half of the screen and the player moved their finger on the blank lower half of the tablet to control the cursor, isolating visuospatial processing and working memory and simulating the need to track the ball while monitoring peripheral cues (e.g., teammates’ positions). The fourth condition was a non-standard split screen cue reversal condition in which the target and cursor again appeared on the top half of the screen and the player moved their finger in the opposite direction of the target on the lower half of the tablet to control the cursor reflecting complex in-match decision-making under pressure, such as executing a pass while evading a defender.

### Data analysis

Head impact exposure rates were calculated as the total number of head impacts divided by the total number of events, with an event being defined as a single practice or match. Head impacts with a peak linear acceleration (PLA) below 7 g were excluded from analysis to align with relevant literature on head impacts in youth soccer players [6]. Goalkeeper impacts were included when PLA exceeded 7 g, as recorded by IMMs during dynamic play (e.g., ground contact during dives or aerial challenges in the box). Cognitive function was examined through the analysis of the brain outcome measures using a custom MATLAB program (MathWorks, Natick, MA, USA) to extract the outcome measures from the raw collected tablet data.

Five outcome measures from each trial of the CMI tasks were used to assess cognitive function: peak finger velocity, absolute error, reaction time, full path movement time, and direction reversal errors. The raw data were processed and these five outcome measures were extracted for each trial in all CMI tasks and examined using a linear mixed effects model. Peak velocity (PV) was the highest velocity (millimetres/millisecond; mm/ms) achieved by the participant’s finger during each trial. Absolute error (AE) was defined as the endpoint accuracy and was determined as the distance between the average initial movement endpoint (defined as the first point beyond the central start circle at which the velocity dropped below 10% peak velocity) for each target location and the actual target position (defined by the x and y coordinates at the center of the target). Reaction time (RT) was calculated as the time interval (milliseconds; ms) from when the peripheral target appeared to when the participant’s finger reached a velocity that was 10% of the peak velocity of the trial [25]. The full path movement time (PMT) was calculated as the amount of time in milliseconds from when the participant initiated movement from the center target to when they reached the correct peripheral target and their movement velocity fell below 10% of the peak velocity at the target. Lastly, the direction reversal errors (DR) were calculated as a deviation of more than ± 45° from the center line of the center target to the correct peripheral target within the first half of the movement [25].

Invalid trials were removed from analysis. Trials were considered invalid if the participant did not keep their finger at the center while waiting for the target to appear, left the center less than 150 ms after the target appeared, left the center more than five seconds after the target appeared, did not hold their finger on the target for 500 ms (swiped through it), or took more than ten seconds to reach the target.

### Statistical analysis

All statistical analyses were completed using R (v4.4.0) [39,40]. Descriptive statistics for total head impacts by player position and header scenario are reported as mean and standard deviation (SD).

Linear mixed-effects models were used to examine the effect of head impacts on the five cognitive outcome measures throughout a season. The interaction between cumulative head impacts and player position were entered as fixed effects and participant, CMI testing point (1–6), and CMI trial number were entered as random effects. Separate models were created for each CMI condition. All linear models were visually examined using residual plots, histograms, and quantile-quantile plots to ensure the model’s validity and reliability. All mixed-effects models were created using the lmerTest package (v3.1.3) [41]. The lmerTest package was used to run an analysis of variance on the linear mixed-effects models. An alpha level was set at *p* ≤ 0.05 and multiple comparison error correction using the Benjamin-Hochberg procedure was applied to control the False Discovery Rate for all tests [42].

## Results

### Participants

Two forwards, nine midfielders, five defenders, and two goalkeepers were equipped with IMMs (18 players). One player had a history of one previous concussion (occurring more than 12 months before enrollment) and one player had a history of obsessive-compulsive disorder. All players participated the whole season and no players removed their consent or dropped-out from the study.

### Heading exposure

Head impact data was recorded for 23 matches and 49 practices throughout this study in which participants attended an average of 17.7 (range: 1–23) matches and 35.1 (range: 10–48) practices. A total of 99 out of 108 expected CMI testing sessions (six sessions for each of the 18 participants) were completed throughout the season. Participants attended an average of 5.5 (range: 2–6) of a possible six CMI testing sessions. A total of 1089 head impact events were recorded for Team A players between practices and matches, of this 37.3% (n = 406) occurred during matches and 62.7% (n = 683) occurred during practices. On average, participants experienced a head impact exposure rate of 0.9 ± 0.7 impacts/event. Players experienced an average of 1.0 ± 1.0 head impacts per match and 0.9 ± 0.8 head impacts per practice, resulting in a median of 43 (IQR: 20–67) head impacts per player over the full season.

**Fig 2:**
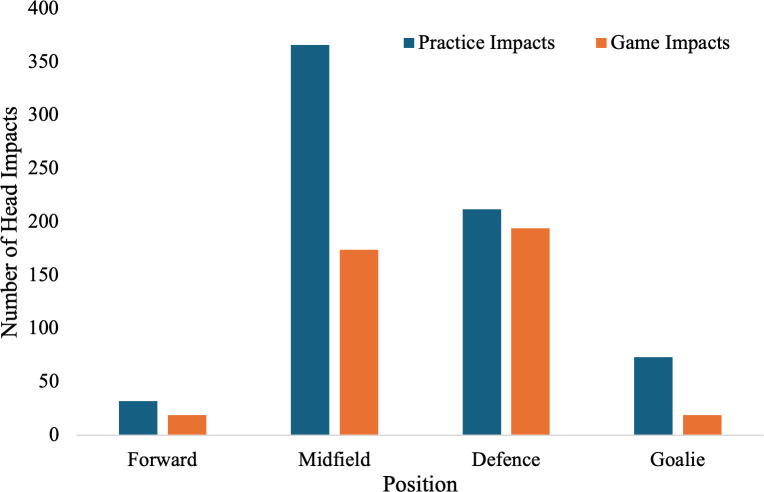
Team A head impacts by player position. Comparison of the number of practice and match head impacts by player position for Team A.

In terms of position, the midfielders experienced the highest number of head impacts (53.6%) during practices with defensive players experiencing the second highest number of head impacts (31.0%). During matches, defensive players experienced the most head impacts (47.8%) followed by midfielders (42.9%, Fig 2).

**Fig 3:**
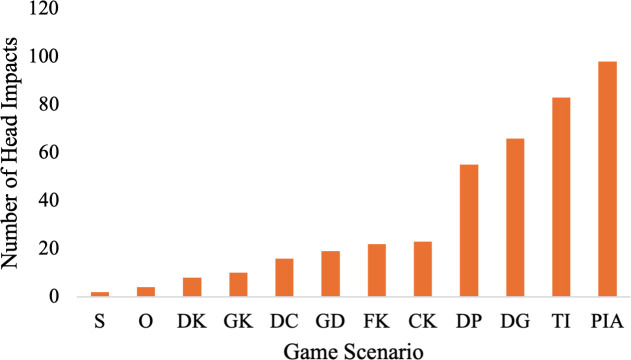
Total team A match head impacts by header scenario. Shot (S). Other (O). Drop Kick (DK). Goal Kick (GK). Defensive Clearing (DC). Goalkeeper Dive (GD). Free Kick (FK). Corner Kick (CK). Deflection off Player (DP). Deflection off Ground (DG). Throw-in (TI). Pass in the air (PIA).

**Fig 4:**
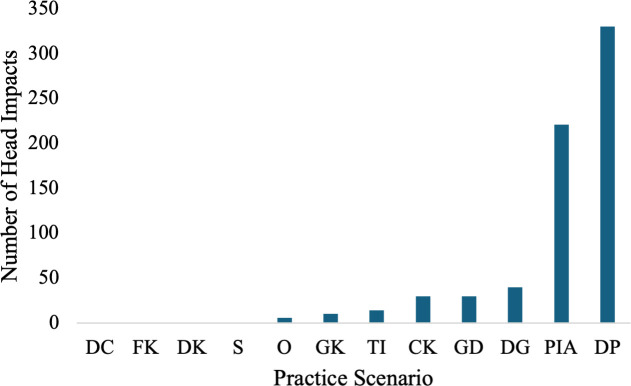
Total team A practice head impacts by header scenario. Defensive Clearing (DC). Free Kick (FK). Dropkick (DK). Shot (S). Other (O). Goal Kick (GK). Throw-in (TI). Corner Kick (CK). Goalkeeper Dive (GD). Deflection off Ground (DG). Pass in the air (PIA). Deflection off Player (DP).

In terms of scenario, passes in the air (24.1%) and throw-ins (20.4%) were the header scenarios which resulted in the most head impacts (Table 1, Fig 3). Head impacts during practices most frequently resulted from deflections off players (48.3%) and passes in the air (32.4%, Fig 4).

**Table 1:** Total match head impacts by header scenario.

Total Head Impacts Recorded	Team A n = 406 (%)	Team B n = 362 (%)	Overall n = 768 (%)
***Header Scenario***			
Corner Kick	23 (5.7%)	23 (6.4%)	46 (6.0%)
Defensive Clearing	16 (3.9%)	11 (3.0%)	27 (3.5%)
Deflection off Ground	66 (16.3%)	87 (24.0%)	153 (19.9%)
Drop Kick	8 (2.0%)	7 (1.9%)	15 (2.0%)
Deflection off Player	55 (13.5%)	53 (14.6%)	108 (14.1%)
Free Kick	22 (5.4%)	24 (6.6%)	46 (6.0%)
Goalkeeper Dive	19 (4.7%)	0 (0.0%)	19 (2.5%)
Goal Kick	10 (2.5%)	4 (1.1%)	14 (1.8%)
Other	4 (1.0%)	0 (0.0%)	4 (0.5%)
Pass in Air	98 (24.1%)	105 (29.0%)	203 (26.4%)
Shot	2 (0.5%)	0 (0.0%)	2 (0.3%)
Throw-in	83 (20.4%)	48 (13.3%)	131 (17.1%)
***Position***			
Forward	19 (4.7%)	47 (13.0%)	66 (8.6%)
Midfielder	174 (42.9%)	171 (47.2%)	345 (44.9%)
Defender	194 (47.8%)	144 (39.8%)	338 (44.0%)
Goalkeeper	19 (4.7%)	0 (0.0%)	19 (2.5%)

**Fig 5:**
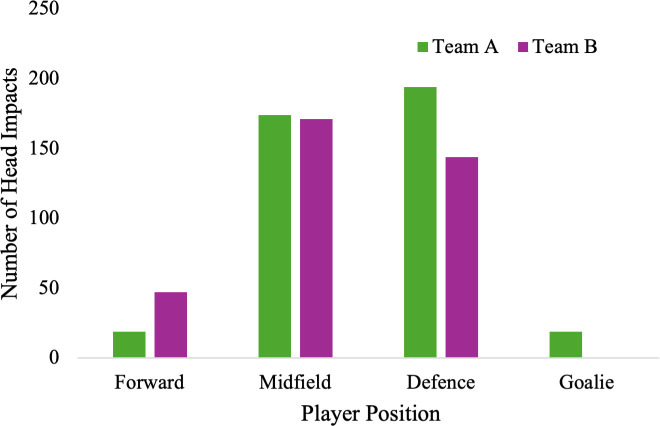
Total match head impacts by player position. Comparison of the number of head impacts by player position for Team A and Team B.

Head impacts for Team B varied slightly from Team A, as most head impacts frequently occurred from passes in the air (29%) and deflections off the ground (24%, Table 1). Compared to Team A, the Team B midfielders experienced the most head impacts (47.2%) followed by defenders (38.9%, Fig 5).

### Cognitive-motor integration tasks

**Fig 6:**
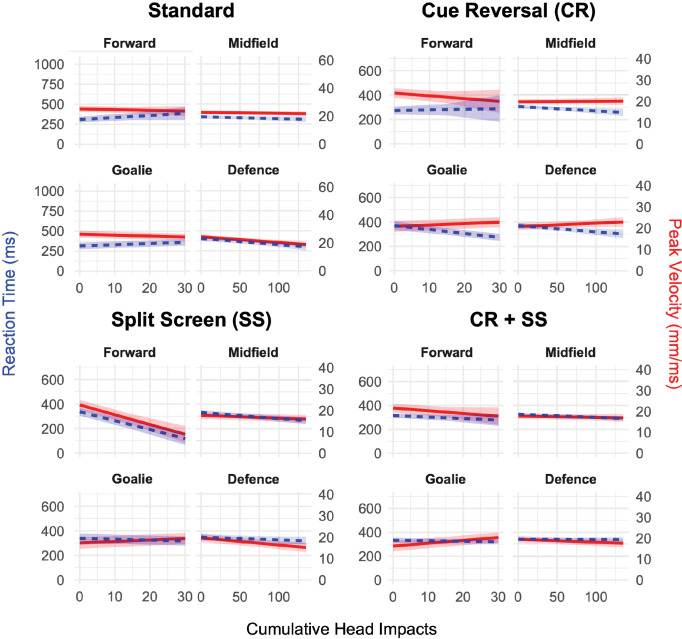
Reaction time and peak velocity across four cognitive-motor integration conditions during a youth soccer season across all athletes in the study. Error bars represent standard error of the mean. Blue lines and shading represent reaction time (milliseconds) interpreted using the left scale, while red lines and shading represent peak velocity (millimeters per millisecond) interpreted using the right scale. Data are shown across cumulative head impacts experienced by the athlete, with separate panels for each condition and faceted by player position. Forwards and goalkeepers accumulated a maximum of 19 head impacts in the dataset; therefore, their model predictions were restricted to 30 impacts to avoid overinterpretation of extrapolated values.

The linear models did not violate the assumptions of linearity, homoskedasticity, or normality of residuals, so the data were not transformed. Sensitivity analyses showed that including concussion history as a fixed effect did not meaningfully alter results of any executed analyses. Measures of peak velocity for the different player positions in the third CMI condition were significantly influenced by cumulative head impacts (F(3, 1097) = 8.37, *p* < 0.001), with a main effect of cumulative head impacts (F(1, 967) = 8.13, *p* = 0.004). For every head impact experienced by a forward, their peak velocity decreased by 0.46 mm/ms (95% CI, −0.69, −0.19). For every head impact experienced by a midfielder, defender, and goalkeeper, their peak velocity increased by 0.44 mm/ms, 0.42 mm/ms, and 0.53 mm/ms respectively, compared to forwards. Cumulative head impacts were not significantly associated with changes in peak velocity by player position in condition one (F(3, 325) = 1.89, *p* = 0.13) or condition two (F(3, 330) = 0.69, *p* = 0.56). The interaction of cumulative head impacts and position was significantly associated with changes in peak velocity in condition four (F(3, 863) = 5.49, *p* = 0.001, Fig 6), and there were no main effects of position (F(3, 17) = 1.37, *p* = 0.29) or cumulative head impacts (F(1, 428) = 0.01, *p* = 0.94).

Measures of absolute error for the different player positions in the first CMI condition were significantly influenced by cumulative head impacts (F(3, 88) = 6.26, *p* < 0.001), with a main effect of position (F(3, 16) = 8.75, *p* = 0.001). At baseline, the defenders’ absolute error was 11 mm higher when compared to forwards (*p* < 0.001*)*. For every head impact experienced by a defender, their absolute error decreased by 0.55 mm (95% CI, −0.93, 4.90x10^-3^) compared to forwards. Head impacts did not significantly influence absolute error for forwards, midfielders, or goalkeepers in the first CMI condition. Cumulative head impacts were not significantly associated with changes in absolute error by player position in condition two (F(3, 208) = 2.29, *p* = 0.08) or condition three (F(3, 884) = 1.77, *p* = 0.15). The interaction of cumulative head impacts and position was significantly associated with changes in absolute error in condition four (F(3, 768) = 3.79, *p* = 0.01), however, there were no main effects of position (F(3, 18) = 0.72, *p* = 0.55) or cumulative head impacts (F(1, 379) = 0.82, *p* = 0.36).

Measures of reaction time for the different player positions in the third CMI condition were significantly influenced by cumulative head impacts (F(3, 583) = 6.36, *p* < 0.001), with a main effect of position (F(1, 198) = 24.62, *p* < 0.001). For every head impact experienced by a midfielder, their reaction time increased by 6.75 ms (95% CI, 3.07, 9.60) compared to forwards. For every head impact experienced by a defender, their reaction time increased by 7.03 ms (95% CI, 3.30, 9.80) compared to the forwards. For every head impact experienced by a goalkeeper, their reaction time increased by 6.52 ms (95% CI, 2.80, 9.40) compared to the forwards. Cumulative head impacts were not significantly associated with changes in reaction time by player position in condition one (F(3, 366) = 2.29, *p* = 0.078), or condition four (F(3, 519) = 0.69, *p* = 0.56). There was no significant interaction of cumulative head impacts with position changes in reaction time (F(3, 183) = 1.96, *p* = 1.12), or for the main effect of cumulative head impacts (F(1, 244) = 0.70, *p* = 0.40), however, the main effect of position was significantly associated with changes in reaction time in condition two (F(3, 27) = 3.16, *p* = 0.04).

Repetitive head impacts did not significantly influence path movement time or direction reversals for different positions in any of the four CMI task conditions.

Notably, technical issues led to the exclusion of 2762 (37.0%) CMI task trials from analysis. Of these, 1595 (21.4%) trials were excluded due to either improper finger contact with the tablet or the tablet failing to register participant input, while the remaining 1167 (15.6%) trials were excluded due to tablet malfunctions.

## Discussion

This study analyzed and characterized total season head impacts of elite youth male soccer players and examined the effects of repetitive head impacts on their cognitive functioning. This study measured position-specific CMI deficits, with midfielders, defenders, and goalkeepers exhibiting deficits in reaction time and increases in peak velocity in the split-screen CMI task that decoupled visual input and motor execution. Defenders showed the greatest cognitive decline, contrary to our hypothesis that midfielders would exhibit the most impairment, reflecting defenders’ higher repetitive head impact exposure. Additionally, 62.7% of head impacts in a single season occurred during practices, with midfielders experiencing the highest head impact exposure while defenders experienced the highest match head impact exposure. These findings highlight that cognitive consequences of repetitive head impacts, alongside impact frequency, are essential to understanding sport-related risks. The results support our hypothesis that repetitive head impacts impair cognitive function throughout a single soccer season, with deficits modulated by position-specific exposure patterns.

The athletes in this study experienced more head impacts during practices compared to matches which is consistent with youth female soccer athletes [43–46]. However, collegiate soccer studies report that both female and male players experience more than six times as many head impacts in matches relative to practices across a single season [9,47]. This inconsistency between youth and collegiate soccer may reflect age-dependent coaching strategies that modulate head impact exposure through variations in drill selection and practice intensity [48].

For example, the Team A coach prioritized heading-specific drills, dedicating approximately half of weekly practices to match scenarios involving deliberate heading practice, consistent with the developmental need to teach proper heading mechanics. This emphasis may stem from the athletes’ developmental stage, as younger players require more technical training to master proper heading mechanics. Similarly to previous studies, most head impacts originated from long-range passes and throw-ins [7–9]. However, defenders – rather than midfielders, as previously reported [49] – exhibited the highest per-position impact frequency [42]. We posit this divergence arises from Team B’s predominant use of a kick-and-run tactical approach, wherein their defenders and midfielders frequently launched aerial balls behind Team A’s defensive line, forcing Team A defenders to intercept via headers. Consequently, Team B forwards also demonstrated elevated head impact exposure relative to Team A forwards, likely due to their role in receiving these long passes.

The observed contrast in head impact distribution between teams underscores coaching philosophy differences. While Team B relied on direct, high-ball tactics, Team A emphasized spatial awareness through short, ground-based passing and off-ball movement – a strategy inherently limiting aerial challenges and in-air turnovers during matches.

The defenders’ higher absolute error by 11 mm at baseline compared to forwards likely contributed to their subsequent improvement as they were more susceptible than the other positions to improvements in absolute error due to a practice effect. This contrasts with recent work where previously concussed male athletes exhibited a speed-accuracy compensation with maintained accuracy despite reduced speed [50].The discrepancy may be attributed to Team A’s low head impact exposure rate compared to prior youth soccer cohorts [3,4,51]. Therefore, baseline differences in absolute error and practice effects may collectively explain the defenders’ reduced absolute error.

Repetitive head impacts selectively impair reaction time in youth soccer players during CMI task condition three (split-screen visuomotor mapping), with midfielders, defenders, and goalkeepers showing significant delays compared to forwards. This position-specific effect aligns with their higher head impact exposure and suggests that task complexity influences sensitivity to repetitive head impacts. Condition three’s split-screen design – which decouples visual input (target/cursor on the top half) from motor execution (finger movement on the bottom half) – likely disrupts visuomotor integration, a process requiring precise coordination between parietal and premotor cortices [52]. The observed slowing implies an impaired ability to translate spatial visual cues into appropriate motor actions, a deficit previously documented in professional hockey players during post-concussion recovery [19]. This raises concerns that repetitive head impacts without diagnosed concussion may produce comparable cognitive-motor deficits, blurring the distinction between non-concussive and concussive injury mechanisms.

The lack of significant associations in conditions one (simple reaction) and four (split-screen + reversal) may reflect task-specific resilience. Condition one’s simplicity (direct stimulus-response mapping) likely engages automated motor pathways less susceptible to mild neurophysiological disruption. Conversely, condition four’s added cognitive load (cue reversal + split-screen) might introduce ceiling effects or compensatory strategies (e.g., conscious error correction), masking subtle deficits. This aligns with studies that showed moderately complex tasks best reveal subclinical cognitive-motor deficits after repetitive head impacts [53,54].

Forwards, who experienced fewer head impacts than other positions, exhibited reaction time improvements over the season, potentially reflecting exercise-induced neuroplasticity [55,56]. Aerobic activity, such as repeated sprinting inherent to forward play, enhances executive function and motor learning, which may offset low-level repetitive head impact effects. In contrast, midfielders and defenders, who engage in frequent aerial challenges and physical collisions, lack this protective buffer due to their higher impact burden. Goalkeepers’ significant delays may reflect differences in impact mechanisms (e.g., ground contact during dives impacting rotational acceleration), which was not examined in this analysis.

The magnitude of this slowed reaction time is particularly concerning: the per-impact reaction time increase observed in this study was approximately 1200 times greater than the saccadic latency impairment reported per head impact in adult university football players [57]. This disparity persists despite youth athletes sustaining far fewer head impacts (1.0 per match versus 37.1 in football [58]), underscoring the heightened vulnerability of adolescents during this sensitive period of cognitive development. The brain continues to develop into early adulthood with the area of the brain responsible for executive function reaching peak gray matter volume at the age of 12 [13]. Repetitive head impacts experienced during this time could interfere with the optimal development of this area and result in executive function impairments that worsen cognitive performance later in life, as seen in adult football players who experienced head impacts before the age of 12 [15]. These reaction time deficits may have meaningful functional consequences, as slower responses could impair situational awareness and increase injury risk during play [59].

Current safety policies, such as Ontario Soccer’s, United States Soccer’s, and England Football’s heading restrictions (full prohibition for U11 players; limited to 5 headers/week in U12-U13 training [60–62]), were implemented to mitigate head impact exposure. However, our results demonstrate measurable cognitive motor impairments even under these limits, suggesting that existing [60–62] guidelines may not sufficiently protect adolescent athletes. Recent trials have examined the effects of banning heading in U12 soccer aiming to gain evidence to encourage the Internation Football Association Board to ban heading for U12 and below players entirely [63]. Given the sensitivity of the developing brain to repetitive head impacts, governing bodies should reevaluate whether age restrictions should be extended to older youth cohorts. In parallel, programs such as *Get aHEAD Safely in Soccer*, developed by United Soccer Coaches, offer practical guidance on how to safely teach heading techniques using lighter balls and age-appropriate drills for players aged 11–13 [64]. Increasing awareness and implementation of such programs – particularly during practices, where 62.7% of head impacts occurred in our study – could serve as an important step toward reducing cumulative head impact exposure in youth soccer.

Athletes with a higher level of experience with sports that require complex visual-motor integration have a better ability to compensate for the cognitive deficits experienced from a concussion than their less experienced peers [38]. As such, the elite athletes examined in this study may have a higher motor reserve [38], allowing them to maintain their CMI performance while accumulating head impacts in the absence of a concussion. Accordingly, it would be interesting to examine the effects of repetitive head impacts on recreational youth athletes who lack this motor reserve.

This study had limitations that should be acknowledged. Data collection focused on two weekly practices identified through consultation with coaching staff as most likely to involve heading drills and head impact scenarios. Additionally, select players participated in all-star tournaments where head impact exposure was not measured. Accordingly, the reported head impact frequencies for Team A likely represent a conservative estimate of total exposure. For Team B (Team A’s opponents), head impacts were only recorded during matches against Team A, excluding practices and other matches. This limited sampling likely results in underreported total head impact exposure for Team B players. While initial adult-sized boil-and-bite IMMs led to some missed impacts during the first week (before custom-fitted devices were provided), subsequent data loss was minimal. Minor missed head impact data occurred due to player non-compliance, incorrect IMM positioning, and isolated instances of device damage. This study only included one participant that had experienced a prior concussion, so we were not able to directly evaluate the effect of concussion history. There were six CMI testing sessions per athlete throughout the season; of the possible 108 CMI testing sessions, 99 sessions were completed which represents a 92% completion rate. While tablet registration errors prevented recording of some individual trials, the use of linear mixed-effects modeling increased confidence in the results by appropriately accounting for these missing data.

In terms of cognitive-motor integration tasks, the lack of change in peak finger velocity in CMI task conditions one, two, and four in response to repetitive head impacts may be due to a practice effect. A practice effect indicates that participants were able to learn explicitly and implicitly to improve their performance, which was previously measured during CMI tasks [65]. Our study did not include a CMI control group due to the use of a convenience sample, so the practice effect during the CMI tasks was not measured. Furthermore, our results may be underpowered, explaining the unchanged peak finger velocity in CMI conditions one, two, and four.

Since this study was restricted to a single elite youth male soccer team, the results are not generalizable to all levels of play, sex, and age groups. Furthermore, individual playing and coaching styles in other sports can influence head impact exposure [51], and similar effects have been observed in soccer [48].

## Conclusions

This study demonstrates that within a single soccer season, even limited repetitive head impacts were associated with measurable changes to the cognitive function of elite youth male soccer players. While total header counts were low which reflects current age-specific restrictions, there are three findings that warrant attention. The first being that a majority of impacts occurred during preventable practice drills rather than in matches. Additionally, the cognitive effects measured were disproportionate to the exposure frequency which reflects the vulnerable stage of neurodevelopment youth athletes are in. It is also important to note the positional differences in head impact rates; while modest, this suggests that alterations to coaching strategies could further minimize head impact exposures. These results underscore the need to reevaluate whether soccer heading policies for U12-U13 players represent an acceptable margin of risk given the observed cognitive effects. Future research should expand to broader populations to include different levels of competition, sexes, and ages, as well as incorporate non-impact control groups to better isolate repetitive head impacts effects from training adaptions.
